# Update and advances in the role of Nursing in prescribing preventive treatment for tuberculosis

**DOI:** 10.1590/0034-7167.2025780601

**Published:** 2025-10-20

**Authors:** Ricardo Alexandre Arcêncio, Pedro Fredemir Palha, Fernanda Dockhorn Costa Johansen, Ethel Leonor Noia Maciel

**Affiliations:** I Universidade de São Paulo, Ribeirao Preto School of Nursing. Ribeirao Preto, Sao Paulo, Brazil; II Brazilian Tuberculosis Research Network, REDE-TB. Rio de Janeiro, Rio de Janeiro, Brazil; III Brazilian Ministry of Health, General Coordination of Tuberculosis Surveillance, Endemic Mycoses and Non- Tuberculous Mycobacteria. Brasília, Federal District, Brazil.; IV Tuberculosis Surveillance, Endemic Mycoses and Non- Tuberculous Mycobacteria. Brasília, Federal District, Brazil.

The Editorial published by REBEn by Arcêncio, Palha and Maciel ^(1)^ points to the potential of including nurses in Tuberculosis Preventive Treatment (TPT) in order to expand coverage in the country. Since the publication of the Editorial, many advances have taken place in Brazil which have led to the urgent need for a new Editorial, with the main developments.

It must be said that tuberculosis (TB) imposes a heavy burden on Brazilian public health, especially among vulnerable populations. Although it is a preventable and curable disease, it remains one of the main causes of death from infectious diseases in the country.

In this scenario, Tuberculosis Infection (TBI) plays a central role in the dynamics of TB transmission, as it represents a hidden reservoir of *Mycobacterium tuberculosis* in the population. Fighting this silent phase of the disease, through timely detection and treatment, is an indispensable strategy for interrupting the cycle of illness and transmission.

The diagnosis of Tuberculosis Infection (TBI) still represents a major bottleneck in tuberculosis control actions in Brazilian health services, which limits the early identification of cases and compromises preventive strategies. However, nursing professionals have immense potential to act in this field, especially in primary care, where their constant presence and bond with the community can be decisive in expanding access to screening and diagnosis of TBI, and it is essential to invest in strengthening the role of nursing in tackling latent tuberculosis.

Nursing’s role, particularly in prescribing preventive treatment for ITB^(2)^, needs to be recognized as an essential public health policy. The decision to authorize nurses to prescribe this treatment, in accordance with guidelines from the Ministry of Health (Information Note No. 4/2024-CGTM/DATHI/ SVSA/MS)^(3)^, represents not only an expansion of attributions, but recognition of the clinical competence and social commitment of the category.

Brazilian nursing, historically positioned on the front lines of care, plays a leading role in the longitudinal monitoring of users of the Unified Health System (SUS)^(4)^. It is this continuous contact with communities that allows nurses to identify early on individuals at increased risk of progression from ITB to the active form, such as people living with HIV, children under five in contact with bacilliferous cases and people deprived of their liberty. Early prescription of preventive treatment not only reduces the risk of illness, but is also a concrete action for health equity.

The incorporation of the ITB prescription into nurses’clinical practice^(2)^ is supported by scientific evidence and by national guidelines that indicate clear flowcharts for screening, testing (using the tuberculin test and interferon-gamma release assays – IGRA) and starting preventive treatment. Strengthening this practice requires a structured policy of training, supervision and institutional support. This is a strategic move to expand access to care, reduce losses in the prevention cascade and reaffirm the role of the SUS as a model of comprehensive care.

Robust studies indicate that the role of nurses in prescribing and monitoring TPT is important not only in terms of expanding access, but above all improving adherence to therapy ^(5)^. Countries have been expanding the role of nurses, especially in response to the recommendations of the World Health Organization. In the United States, Canada, Australia, the United Kingdom, New Zealand and South Africa, nurses can prescribe TPT according to national and state protocols.

A study in the United States^(5)^ with nurses and homeless people showed an increase in the completion rate from 67% to 85%, making progress on important issues such as drug addiction, mental health and harm reduction. Studies in Spain^(6)^ also showed 88.3% completion of preventive therapy in transplanted people, and showed only 1 case of post-transplant TB out of 1426 people being followed up.

The combination of the strategy with portable technologies, such as X-ray, has been a potentiality in terms of TB detection to overcome the bottlenecks we have, such as the waiting time for X-ray, greater readiness and immediacy of TPT, every minute that is lost is more time and opportunity for the bacillus.

However, progress in TPT requires structural investments. The launch of continuing education, which qualifies the strategies for monitoring the quality of care provided, is an important factor. One example is the initiative of the CGTM of the DATHI/SVSA, which in 2025, in partnership with the Brazilian Tuberculosis Research Network – REDE-TB and the Ribeirao Preto Nursing School of the University of São Paulo, is launching an online course for nurses linked to management and care.

The course includes key issues such as the epidemiology of tuberculosis, clinical protocols, screening flowcharts, the use of diagnostic tests (tuberculin test and IGRA), clinical protocols, relevant information on X-rays and the management of adverse effects. A total of 284 nurses were selected, including representatives from all states and the Federal District, who will receive 40 hours of online training, distributed between 12 modules, and a formative evaluation model, in terms of knowledge and attitudes towards TPT practices. The sustainability of the continuing education policy is fundamental for recognizing the work of nursing as essential to the national response to TB.

Nursing’s role in prescribing preventive treatment for tuberculosis represents a transformative milestone for the SUS and for Brazilian public health. More than an increase in attributions, it is a recognition of the technical capacity, ethical commitment and centrality of nurses in the comprehensive care of vulnerable populations. Investing in the qualification and autonomy of nursing professionals results in more agile responses and satisfactory health outcomes, expanding access to care and reducing health inequalities. When nursing is positioned as a strategic agent for tuberculosis prevention, it reaffirms its transformative role in the SUS and is an important step in building a future free of the disease, but it is necessary to recognize the value of nurses’ work in the SUS.
