# Supplementary material for: Update and advances in the role of Nursing in prescribing preventive treatment for tuberculosis
Source: Rev Bras Enferm. 2025 Oct 20;78(6):e780601. doi: 10.1590/0034-7167.2025780601 (PMC12536995; doi:10.1590/0034-7167.2025780601)
Supplement: Translation

# Translation: Atualização e avanços no protagonismo da Enfermagem na prescrição do tratamento preventivo da tuberculose

**DOI:** 10.1590/0034-7167.2025780601pt

**Authors:** Ricardo Alexandre Arcêncio, Pedro Fredemir Palha, Fernanda Dockhorn Costa Johansen, Ethel Leonor Noia Maciel

**Affiliations:** I Universidade de São Paulo, Escola de Enfermagem de Ribeirão Preto. Ribeirão Preto, São Paulo, Brasil.; II Rede Brasileira de Pesquisas em Tuberculose, REDE-TB. Rio de Janeiro, Rio de Janeiro, Brasil.; III Ministério da Saúde, Coordenação-Geral de Vigilância da Tuberculose, Micoses Endêmicas e Micobactérias Não Tuberculosas. Brasília, Distrito Federal, Brasil.; IV Universidade Federal do Espírito Santo, Departamento de Enfermagem. Vitória, Espírito Santo, Brasil.

O Editorial publicado pela REBEn de Arcêncio, Palha e Maciel^(1)^ acena sobre as potencialidades da inclusão de enfermeiros na Tratamento Preventivo da Tuberculose (TPT) no sentido de ampliação da cobertura no país. Desde a publicação do Editorial, muitos avanços ocorreram no Brasil que convergiram na necessidade de um novo Editorial, com os principais desdobramentos. A tuberculose (TB) impõe um pesado fardo à saúde pública brasileira, especialmente entre populações vulnerabilizadas. Embora seja uma doença evitável e curável, permanece como uma das principais causas de morte por doença infecciosa no país.

No cenário, a Infecção por Tuberculose (ITB) assume papel central na dinâmica de transmissão da TB, ao representar uma reserva oculta do *Mycobacterium tuberculosis* na população. Combater a fase silenciosa da doença, por meio da detecção e tratamento oportuno, é uma estratégia indispensável para interromper o ciclo de adoecimento e transmissão.

O diagnóstico da Infecção por Tuberculose (ITB) ainda representa um importante gargalo nas ações de controle da tuberculose nos serviços de saúde do Brasil, o que limita a identificação precoce das pessoas acometidas e compromete estratégias preventivas, contudo os profissionais de enfermagem têm um potencial imenso de atuação nesse campo, especialmente na Atenção Primária à Saúde, onde sua presença constante e vínculo com a comunidade podem ser decisivos para ampliar o acesso ao rastreamento e diagnóstico da ITB, sendo fundamental investir no fortalecimento do papel da enfermagem no enfrentamento da tuberculose infecção.

A atuação da Enfermagem, particularmente na prescrição do tratamento preventivo da ITB^(2)^, precisa ser reconhecida como uma política de saúde pública essencial. A decisão de autorizar enfermeiros a prescreverem esse tratamento, conforme orientações do Ministério da Saúde (Nota Informativa nº 4/2024-CGTM/DATHI/SVSA/MS)^(3)^ representa não apenas uma ampliação de atribuições, mas o reconhecimento da competência clínica e do compromisso social da categoria.

A Enfermagem brasileira, historicamente posicionada nas linhas de frente do cuidado, é protagonista no acompanhamento longitudinal dos usuários do Sistema Único de Saúde (SUS) ^(4)^. É esse contato longitudinal com as comunidades que permite ao enfermeiro identificar precocemente indivíduos com risco aumentado de progressão da ITB para a forma ativa, como pessoas vivendo com HIV, crianças menores de cinco anos em contato com casos bacilíferos e populações privadas de liberdade. A prescrição precoce do tratamento preventivo não apenas reduz o risco de adoecimento, como também é uma ação concreta de equidade em saúde.

A incorporação da prescrição da ITB à prática clínica do enfermeiro^(2)^ é respaldada por evidências científicas e por diretrizes nacionais que indicam fluxogramas claros para triagem, testagem, por meio da prova tuberculínica e dos ensaios de liberação de interferon-gama – IGRA e início do tratamento preventivo. O fortalecimento dessa prática requer uma política estruturada de formação, supervisão e apoio institucional. Trata-se de um movimento estratégico para ampliar o acesso ao cuidado, reduzir perdas na cascata de prevenção e reafirmar o papel do SUS como modelo de atenção integral.

Estudos robustos apontam que a atuação do enfermeiro na prescrição e no acompanhamento do TPT é importante não só no sentido de ampliar o acesso, mas sobretudo melhorar a adesão à terapêutica^(5)^. Países vêm ampliando a atuação do enfermeiro, especialmente em resposta às recomendações da Organização Mundial da Saúde. Nos Estados Unidos, Canadá, Austrália, Reino Unido, Nova Zelândia, África do Sul também, na lógica da ampliação de escopo de prática, enfermeiros podem prescrever TPT, segundo protocolos nacionais e estaduais.

Estudo nos Estados Unidos^(5)^, com enfermeiros e pessoas em situação de rua, evidenciou uma elevação da taxa de completude de 67% para 85% avançando em questões importantes como drogadição, saúde mental, e redução de danos. Estudos na Espanha^(6)^ também evidenciou 88,3% de conclusão da terapia preventiva em pessoas transplantadas, e mostrou apenas um caso de TB pós-transplantes, em 1426 pessoas em seguimento.

A combinação da estratégia com tecnologias portáteis, como o Raio X, tem sido uma potencialidade no que se refere à detecção da TB para superar os gargalos que temos, como o tempo de espera do Raio X, maior prontidão e imediatez do TPT, cada minuto que se perde, é mais tempo e oportunidade para o bacilo.

No entanto, o avanço do TPT exige investimentos estruturantes. O lançamento da educação permanente que qualifiquem as estratégias de monitoramento da qualidade do cuidado prestado é um fator relevante. Um exemplo é a iniciativa da CGTM do DATHI/ SVSA, que em 2025, em parceria com a Rede Brasileira de Pesquisas em Tuberculose – REDE-TB e com a Escola de Enfermagem de Ribeirão Preto da Universidade de São Paulo, está lançando um curso de formação, destinado a enfermeiros vinculados à gestão e assistência, intitulado “Quali-TPT”.

No referido curso, estão incluídas questões-chave como a epidemiologia da tuberculose, protocolos clínicos, fluxogramas de triagem, uso de testes diagnósticos, protocolos clínicos, informações relevantes sobre o Raio X e manejo de efeitos adversos. Foram selecionados 284 enfermeiros, incluindo representantes de todos os estados e do Distrito Federal, que receberão formação on-line de 40 horas, distribuídos entre 12 módulos, e um modelo da avaliação formativa, no sentido de avaliar conhecimento, atitudes e práticas para o TPT. A sustentabilidade da política de educação permanente é fundamental para o reconhecimento do trabalho da Enfermagem como essencial à resposta nacional à TB. O protagonismo da Enfermagem na prescrição do tratamento preventivo da tuberculose representa um marco transformador para o SUS e para a saúde pública. Mais do que uma ampliação de atribuições, trata-se do reconhecimento da capacidade técnica, do compromisso ético e da centralidade do enfermeiro no cuidado integral às populações vulnerabilizadas. Investir na qualificação e autonomia dos profissionais de Enfermagem resulta em respostas mais ágeis resultados sanitários satisfatórios, ampliando o acesso ao cuidado e reduzindo as iniquidades em saúde. A Enfermagem ao ser posicionada como ator estratégico prevenção da tuberculose, reafirma o seu papel transformador no SUS e passo importante na construção de um futuro livre da doença, todavia é preciso reconhecer o valor do trabalho da enfermagem no SUS.

# Translation: Actualización y avances en el rol de Enfermería en la prescripción del tratamiento preventivo de la tuberculosis

**DOI:** 10.1590/0034-7167.2025780601es

**Authors:** Ricardo Alexandre Arcêncio, Pedro Fredemir Palha, Fernanda Dockhorn Costa Johansen, Ethel Leonor Noia Maciel

**Affiliations:** I Universidade de São Paulo, Escuela de Enfermería de Ribeirão Preto. Ribeirão Preto, São Paulo, Brasil.; II Red Brasileña de Investigación en Tuberculosis, REDE-TB. Río de Janeiro, Río de Janeiro, Brasil.; III Ministerio de Salud de Brasil, Coordinación General de Vigilancia de Tuberculosis, Micosis Endémicas y Micobacterias No Tuberculosas. Brasília, Distrito Federal, Brasil.; IV Universidade Federal do Espírito Santo, Departamento de Enfermería. Vitória, Espírito Santo, Brasil.

El Editorial publicado por REBEn de Arcêncio, Palha y Maciel^(1)^ señala el potencial de la inclusión de enfermeros en el Tratamiento Preventivo de la Tuberculosis (TPT) para ampliar la cobertura en el país. Desde la publicación del Editorial, muchos avances han tenido lugar en Brasil que han llevado a la necesidad urgente de un nuevo Editorial, con las principales novedades.

Hay que decir que la tuberculosis (TB) impone una pesada carga a la salud pública brasileña, especialmente entre las poblaciones vulnerables. A pesar de ser una enfermedad prevenible y curable, sigue siendo una de las principales causas de muerte por enfermedades infecciosas en el país.

En este escenario, la Infección Tuberculosa (ITB) desempeña un papel central en la dinámica de transmisión de la tuberculosis, ya que representa un reservorio oculto de *Mycobacterium tuberculosis* en la población. Combatir esta fase silenciosa de la enfermedad, mediante la detección y el tratamiento oportunos, es una estrategia indispensable para interrumpir el ciclo de enfermedad y transmisión.

El diagnóstico de la infección tuberculosa (ITB) todavía representa un gran cuello de botella en las acciones de control de la tuberculosis en los servicios de salud de Brasil, lo que limita la identificación precoz de los casos y pone en peligro las estrategias preventivas. Sin embargo, los profesionales de enfermería tienen un enorme potencial de acción en este campo, especialmente en la atención primaria, donde su presencia constante y su vínculo con la comunidad pueden ser decisivos para aumentar el acceso a la detección y el diagnóstico de la ITB, y es esencial invertir en el fortalecimiento del papel de la enfermería en el abordaje de la tuberculosis latente.

El papel de la enfermería, especialmente en la prescripción del tratamiento preventivo de la ITB^(2)^, debe ser reconocido como una política esencial de salud pública. La decisión de autorizar a las enfermeras a prescribir este tratamiento, de acuerdo con las directrices del Ministerio de Salud (Nota Informativa nº 4/2024-CGTM/DATHI/SVSA/MS)^(3)^, representa no sólo una ampliación de atribuciones, sino el reconocimiento de la competencia clínica y del compromiso social de la categoría.

La enfermería brasileña, históricamente posicionada en la primera línea de atención, desempeña un papel destacado en el seguimiento longitudinal de los usuarios del Sistema Único de Salud (SUS)^(4)^. Es este contacto continuo con las comunidades que permite a las enfermeras identificar precozmente individuos con mayor riesgo de progresión de la ITB para la forma activa, como personas que viven con VIH, niños menores de cinco años en contacto con casos bacilíferos y personas privadas de libertad. La prescripción precoz del tratamiento preventivo no sólo reduce el riesgo de enfermedad, sino que también es una acción concreta en favor de la equidad sanitaria.

La incorporación de la prescripción de la ITB en la práctica clínica de las enfermeras^(2)^ está respaldada por pruebas científicas y por directrices nacionales que indican diagramas de flujo claros para el cribado, la realización de pruebas (mediante la prueba de la tuberculina y ensayos de liberación de interferón-gamma – IGRA) y el inicio del tratamiento preventivo. Reforzar esta práctica requiere una política estructurada de formación, supervisión y apoyo institucional. Se trata de una medida estratégica para ampliar el acceso a la atención, reducir las pérdidas en la cascada de prevención y reafirmar el papel del SUS como modelo de atención integral.

Estudios sólidos demuestran que el papel de las enfermeras en la prescripción y el seguimiento del TPT es importante no sólo para ampliar el acceso, sino sobre todo para mejorar el cumplimiento terapéutico^(5)^. Los países han ido ampliando el papel de las enfermeras, especialmente en respuesta a las recomendaciones de la Organización Mundial de la Salud. En Estados Unidos, Canadá, Australia, Reino Unido, Nueva Zelanda y Sudáfrica, las enfermeras pueden prescribir TPT según los protocolos nacionales y estatales.

Un estudio realizado en Estados Unidos^(5)^ con enfermeros y personas sin hogar mostró un aumento de la tasa de finalización del 67% al 85% lo que supuso un avance en cuestiones importantes como la drogadicción, la salud mental y la reducción de daños. Estudios realizados en España^(6)^ también mostraron un 88,3% de finalización de la terapia preventiva en personas trasplantadas, y mostraron sólo 1 caso de tuberculosis postrasplante de 1426 personas en seguimiento.

La combinación de la estrategia con tecnologías portátiles, como los rayos X, ha sido una potencialidad en cuanto a la detección de la tuberculosis para superar los cuellos de botella que tenemos, como el tiempo de espera para los rayos X, mayor prontitud e inmediatez de la TPT, cada minuto que se pierde es más tiempo y oportunidad para el bacilo.

Sin embargo, el progreso de la TPT requiere inversiones estructurales. El lanzamiento de la educación continua, que califica las estrategias de seguimiento de la calidad de la atención prestada, es un factor importante. Un ejemplo es la iniciativa del CGTM de la DATHI/SVSA, que, en 2025, en asociación con la Red Brasileña de Investigación en Tuberculosis – REDE-TB y la Escuela de Enfermería de Ribeirão Preto de la Universidad de São Paulo, está lanzando un curso en línea dirigido a enfermeras vinculadas a la gestión y la atención.

El curso incluye temas clave como la epidemiología de la tuberculosis, protocolos clínicos, diagramas de flujo de detección, el uso de pruebas de diagnóstico (prueba de la tuberculina y IGRA), protocolos clínicos, información relevante sobre los rayos X y la gestión de los efectos adversos. Un total de 284 enfermeras fueron seleccionadas, incluyendo representantes de todos los estados y el Distrito Federal, que recibirán 40 horas de formación en línea, distribuidos en 12 módulos, y un modelo de evaluación formativa, en términos de conocimientos y actitudes hacia las prácticas de TPT. La sostenibilidad de la política de formación continua es fundamental para reconocer el trabajo de enfermería como esencial para la respuesta nacional a la TB.

El papel de la enfermería en la prescripción del tratamiento preventivo de la tuberculosis representa un hito transformador para el SUS y para la salud pública brasileña. Más que un aumento de las responsabilidades, es un reconocimiento de la capacidad técnica, el compromiso ético y la centralidad de las enfermeras en la prestación de atención integral a las poblaciones vulnerables. Invertir en la cualificación y autonomía de los profesionales de enfermería se traduce en respuestas más ágiles y resultados sanitarios satisfactorios, ampliando el acceso a los cuidados y reduciendo las desigualdades en salud. Cuando la enfermería se posiciona como agente estratégico para la prevención de la tuberculosis, reafirma su papel transformador en el SUS y es un paso importante hacia la construcción de un futuro libre de la enfermedad, pero es necesario reconocer el valor del trabajo de las enfermeras en el SUS.
